# ﻿A new species of pit-viper from the Ayeyarwady and Yangon regions in Myanmar (Viperidae, *Trimeresurus*)

**DOI:** 10.3897/zookeys.1186.110422

**Published:** 2023-12-13

**Authors:** Kin Onn Chan, Shahrul Anuar, Ananthanarayanan Sankar, Ingg Thong Law, Ing Sind Law, Rasu Shivaram, Ching Christian, Daniel G. Mulcahy, Anita Malhotra

**Affiliations:** 1 Lee Kong Chian Natural History Museum, National University of Singapore, 2 Conservatory Drive, 117377 Singapore, Singapore; 2 School of Biological Sciences, Universiti Sains Malaysia, 11800 Penang, Malaysia; 3 Herpetological Society of Singapore, 12J Sime Road, 288296 Singapore, Singapore; 4 Department of Biological Sciences, National University of Singapore, 16 Science Drive 4, 117558 Singapore, Singapore; 5 Department of Life Sciences, The Natural History Museum, London SW7 5BD, UK; 6 Museum für Naturkunde, Leibniz Institute for Evolution and Biodiversity Science, Invalidenstraße 43, 10115 Berlin, Germany; 7 Molecular Ecology and Evolution at Bangor, School of Natural Sciences, Bangor University, Bangor LL57 2UW, Gwynedd, UK

**Keywords:** Cryptic species complex, mangrove pit-viper, morphology, snake, systematics, taxonomy

## Abstract

In a genomic study by Chan and colleagues, pit-vipers of the *Trimeresuruserythrurus*–*purpureomaculatus* complex from the Ayeyarwady and Yangon regions in Myanmar were demonstrated to be a distinct species based on robust population genetic and species delimitation analyses. Here, we provide morphological characterizations and a formal description of those populations as a new species. The new species, *Trimeresurusayeyarwadyensis***sp. nov.**, is most closely related to *T.erythrurus* and *T.purpureomaculatus* and shares morphological characteristics with both of those species. Some specimens of *T.ayeyarwadyensis***sp. nov.** have green dorsal coloration and no distinct dorsal blotches (a trait shared with *T.erythrurus* but not *T.purpureomaculatus*), while others have dark dorsal blotches (a trait shared with *T.purpureomaculatus* but not *T.erythrurus*). The distinct evolutionary trajectory of the new species, coupled with the lack of obvious morphological differentiation, represents a classic example of the cryptic nature of species commonly found in the *Trimeresurus* group of Asian pit-vipers and underscores the need for data-rich analyses to verify species’ boundaries more broadly within this genus.

## ﻿Introduction

The evolutionary history of Asian pit-vipers of the genus *Trimeresurus* Lacepède, 1804 remains poorly understood and is characterized by phylogenetic uncertainty (Malhotra and Thorpe 2000, 2004a, b; Giannasi et al. 2001; Creer et al. 2003, 2006; Mulcahy et al. 2017; Mallik et al. 2021). Furthermore, complicated phenotypic variation has been a major source of taxonomic confusion (Sanders et al. 2004; Vogel et al. 2004, 2022; Wostl et al. 2016; Mulcahy et al. 2017; Chandramouli et al. 2020; Chen et al. 2020). Within this genus, mangrove pit-vipers of the *T.purpureomaculatus*–*erythrurus* complex exhibit one of the most extreme phenotypic variations that have confounded researchers for decades (Pope and Pope 1933; Smith 1943; Chan et al. 2022).

*Trimeresuruserythrurus* is invariably green with no dorsal markings and is not a mangrove-forest obligate (Deuti et al. 2021). Contrastingly, *T.purpureomaculatus* occurs exclusively in mangroves, has highly variable dorsal coloration (ranging from purplish-gray, yellow, reddish-brown to black, but never green), and typically has distinct dorsal blotches, except for some melanistic populations in Singapore and on Sumatra. Interestingly, the melanistic variant has not been documented north of Singapore. Until recently, this species complex has not been subjected to focused studies primarily because genetic material from the type locality of *T.purpureomaculatus*, which is Singapore, had never been collected.

A topotype of *T.purpureomaculatus* from Singapore was first sequenced by Chan et al. (2022), who provided a preliminary phylogeographic framework that characterized phenotypic variants in association with genetic clades and geographic distribution. Their study revealed a genetically divergent lineage (herein referred to as *T.* sp.) from the Ayeyarwady and Yangon regions in Myanmar that occurs in the intervening region between the distributions of *T.erythrurus* and *T.purpureomaculatus*. Snakes from these regions have green dorsal coloration with varying degrees of blotchiness, which could be considered a blend between the phenotypes of *T.erythrurus* and *T.purpureomaculatus*. Based on mitochondrial DNA divergences and intermediate phenotypic attributes, Chan et al. (2022) hypothesized that the populations from Ayeyarwady and Yangon regions could represent an undescribed species that was well supported as sister to *T.purpureomaculatus*, which has hybridized, or is hybridizing, with *T.erythrurus* and/or *T.purpureomaculatus*.

A subsequent genomic study performed an in-depth investigation into the population genetics of the *Trimeresuruspurpureomaculatus*–*erythrurus* complex with an emphasis on elucidating the species boundaries of *T.* sp. from the Ayeyarwady and Yangon regions in Myanmar (Chan et al. 2023). In that study, gene flow was demonstrated to be present between *T.* sp. and *T.erythrurus* and to a lesser extent, *T.purpureomaculatus* (Chan et al. 2023). Contrasting with the mtDNA, the genomic data placed the new species sister to *T.erythrurus* with strong support. Additionally, a population of *T.erythrurus* from the Pathein District in the Ayeyarwady Region was shown to be highly admixed between *T.erythrurus* and *T.* sp. However, despite genetic admixture, *T.* sp. was unequivocally demonstrated to be an independently evolving lineage that is not conspecific with either *T.erythrurus* or *T.purpureomaculatus* (Chan et al. 2023). In this study, we perform additional analyses and provide a morphological characterization of *T.* sp. from the Ayeyarwady and Yangon regions and its description as a new species.

## ﻿Materials and methods

**Figure 1.**
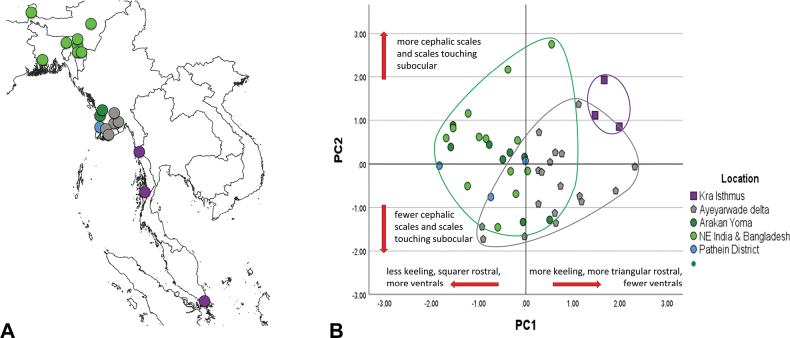
**A** approximate locations of examined specimens grouped and colored according to geographic regions; Purple (Isthmus of Kra) = *Trimeresuruspurpureomaculatus*; green (Arakan Yoma, Northeast India and Bangladesh, and Pathein District, Myanmar) = *T.erythrurus*; gray (Ayeyarwady Delta, Myanmar) = *T.* sp. Overlapping localities are not shown **B** principal component analysis, males and females combined.

A total of 24 female and 26 male vouchered specimens comprising *Trimeresuruserythrurus*, *T.purpureomaculatus*, and *T.* sp. were examined from the holdings of the California Academy of Sciences (**CAS**), University of Florida (**UF**), Naturhistorisches Museum Wien (**NMW**), Museum of Comparative Zoology, Harvard University (**MCZ**), and the Natural History Museum London (**NHMUK**/**BMNH**), as well as anesthetized live specimens from Mizoram (courtesy of HT Lalremsanga, Mizoram University) and Myanmar (courtesy of Michihisa Toriba, Japan Snake Institute). Approximate locations of examined specimens are shown in Fig. 1A. The following mensural and meristic morphological characters were assessed: snout–vent length (**SVL**), tip of snout to tip of tail; tail length (**TaL**), cloaca to tip of tail; head length (**HL**), posterior margin of jaw articulation to tip of snout; head width (**HW**), widest distance across the dorsal surface of head; head width at posterior margin of supraoculars (**HW1**); eye diameter (**ED**), horizontal diameter of eye; eye–nostril–distance (**EYE2NOS**), distance between anterior margin of orbit to posterior margin of nostril; eye–pit–distance (**EYE2PIT**), distance between anterior margin of orbit to posterior margin of pit; nostril–pit–distance (**NOS2PIT**), distance between the nostril and the pit, measured between the outer edges; length of supraocular (**LSUP**) measured on the right side; width of supraocular (**WSUP**), measured on the right side; width of the internasal (**WINTNAS**), measured horizontally on the right side; ventral scales (**VEN**), number of ventral scales; subcaudals (**SC**) number of subcaudal scales; number of supralabials (**SL**) and infralabials (**IL**), average of left and right side; cephalic scales (**CEP**), minimum number of cephalic scales between left and right supraoculars; minimum number of scales between posterior margins of suraoculars (**BTWSUP**); number of scales bordering the supraoculars (**BORSUPOC**), average of right and left scales, not including post- and pre-ocular scales; the number of scales between the nasal scale and the shield bordering the pit anteriorly (**NASPIT**); the number of scales separating the internasal scales (**INTNAS**); rostral shape (**ROST**), the ratio of the dorsal margin of the rostral scale to the ventral margin; the number of paired chin shields between mental scale and first ventral scale (**CHIN**); the number of scales between the edge of the mouth and the ventral scales/chin shields, starting at and including the last sublabial (**VENTEDGE**). We also measured the position of dorsal scale row reductions from 31 to 29 scales (occurring just behind the head), 29–27 scales, 27–25, 25–23, 23–21, 21–19, and 19–17 scales (occurring just before the vent). Each reduction was recorded against the corresponding ventral scale row and converted to a percentage of VEN before analysis. Similarly, the reductions on the tail from 14–12 scale rows, 12–10, 10–8, 8–6, and 6–4 scale rows were measured against subcaudal scales and converted to a percentage of SC before analysis. A few ordinal characters were also included: keeling of body scales (**BSCK**) measured at mid-body; keeling of temporal scales (**KTEMP**); keeling of head scales (**KHEAD**), measured on a scale of 0 (no keeling) to 1.5 (sharp keel present) in 0.5 increments; raw measurements and counts for all examined specimens are listed in Suppl. material 1.

To control bias stemming from sexual dimorphism (Chan and Grismer 2021) while maximizing sample sizes, males and females were analyzed initially using a two-way analysis of variance/co-variance. Specimens were grouped for this purpose using biogeographic criteria into 5 groups: North-East India and Bangladesh (10 males, 7 females), Arakan Yoma (4 males, 4 females), Ayeyarwady delta excluding Pathein District (8 males, 11 females), Pathein District (1 male, 2 females), and Kra Isthmus (3 males, 0 females). Mensural characters were first log-transformed. Levene’s test for homogeneity of variances was performed, and if significant, the Brown-Forsythe alternative to ANOVA was considered instead. Characters showing non-significant between-group variation were not considered further. We performed allometric body-size correction for mensural characters using the Thorpe method (Thorpe 1983). Principal components analysis (PCA) was used to find the best low-dimensional representation of variation in the data to determine whether morphological variation could form the basis of detectable group structure. Unrotated PCs were extracted from the correlation matrix and the number of PCs to be retained was based on examination of the scree plot.

To gain greater clarity on diagnostic characters separating the two geographically proximate and introgressing species, we carried out a discriminant analysis on the same set of characters on each sex separately, but also including sexually dimorphic characters. Variables were entered in a stepwise manner, with the variable that minimized the overall Wilks’ lambda entered at each step, and also included some characters reflecting the prominence of the postocular and lateral stripes: OC (number of scales covered by the postocular white stripe) and SC1 (proportion of first scale row at 50% VEN covered by white area). The minimum partial F-to-enter was 3.84. Leave-out-one classification was performed to determine the robustness of the result. All morphological analyses were performed and visualized using SPSS v. 27.0.

## ﻿Results

Summary statistics of raw mensural and meristic data are presented in Table 1. Two-way ANOVA or ANCOVA showed significant (*P* < 0.05) differences between sexes but not among groups in HL, HW1, and TL. Among-group variation was significant in the following characters: VEN, SC, BSCK, SL, CEP, HW, NASPIT, ED, SOCBORD, ROST, KTEMP, KHEAD, VS%25–23, SC%10–8, SC%8–6. Of these, SC, SL, HW, SC%8–6; ED were also significantly different between sexes. ED showed a significant interaction between group and covariate (HL) and hence was discarded as the difference between within-group slopes invalidates size adjustment. Levene’s test showed significant heteroscedasticity in VEN, SC, CEP, KTEMP, KHEAD, VS%25–23. Meristic characters were tested further using the Brown-Forsythe alternative in one-way ANOVA (for each sex separately) which relaxes the assumption of equal variances between groups, and if they remained significant in either sex, they were retained for further analysis. However, it was not possible to carry out robust tests for KTEMP and KHEADSC as at least one group had zero variance.

**Table 1.** Summary statistics for specimens examined in this study. Snout-vent length and tail length are measured in mm and statistics are calculated from specimens over 350 mm SVL (males) or 400 mm (females) only. Values shown are mean ± standard deviation followed by min–max in parentheses.

	*T.erythrurus*		*T.purpureomaculatus*	*T.* sp.	
	male (*n* = 15)	female (*n* = 13)	male (*n* = 5)	male (*n* = 8)	female (*n* = 11)
SVL	484.1 ± 55.8 (398–582)	634.4 ± 91.6 (478–746)	522.8 ± 57.7 (456–610)	477.9 ± 23.6 (432–500)	647.5 ± 177 (459–935)
TaL	120.9 ± 16.7 (97–146)	102.2 ± 2.32 (57–127)	139.6 ± 13.61 (123–158)	128.3 ± 0.85 (112–135)	112.4 ± 28.6 (84–165)
SL	10.9 ± 0.72 (9–13)	11.7 ± 0.81 (10–13)	10.8 ± 0.91 (9–12)	10.2 ± 0.7 (9–11)	10.9 ± 0.65 (10–12)
IF	12.7 ± 0.68 (12–14)	13.19 ± 1.23 (10–15)	12.9 ± 0.96 (11–14)	12.7 ± 1.04 (11–15)	13.05 ± 0.79 (12–14)
VEN	166.4 ± 5.7 (156–181)	168.4 ± 5.32 (163–180)	161 ± 3.16 (156–164)	161.7 ± 5. 92 (150–170)	165.1 ± 4.89 (157–169)
SC	65.67 ± 3.22 (60–71)	56.4 ± 6.12 (49–68)	73.2 ± 2.77 (69–76)	73.3 ± 1.75 (71–76)	55.0 ± 2.14 (52–58)

The characters that were entered into the PCA with both sexes included after this initial screening step were VEN, BSCK, CEP, NASPIT, SOCBORD, ROST, KTEMP, KHEAD, VS%25–23, and SC%10–8 (sexually dimorphic characters were not included). The resulting graph showed that *T.* sp. could be partly distinguished from *T.purpureomaculatus* (Kra Peninsula) and *T.erythrurus* (NE India and Bangladesh, Arakan Yoma, and Pathein District) on the first two axes. Separation can be observed between *T.purpureomaculatus* and *T.erythrurus* along PC1 (Fig. 1B), largely determined by the degree of keeling on the body, head, and temporal scales, the shape of the rostral scale, and also the reduction from 25 to 23 scale rows occurring further down the body in *T.purpureomaculatus*. However, *T.* sp. overlaps with both *T.purpureomaculatus* and *T.erythrurus* on PC1. The PC2 axis largely distinguishes *T.* sp. from *T.purpureomaculatus* on the basis of possessing a lower number of cephalic scales (CEP) and scales bordering the subocular (SOCBORD), while the position of the scale reduction from 10 to 8 scale rows on the tail (SC%10–8) occurs closer to the vent. Thus, while a combination of these characters can distinguish most specimens of the three species, they may not be diagnostic for all specimens. Note that the Pathein District specimens, which showed substantial genetic introgression between *T.erythrurus* and *T.* sp. (Chan et al. 2023), appear to be morphologically identical to *T.erythrurus* and were grouped with them in the subsequent discriminant analysis.

**Figure 2.**
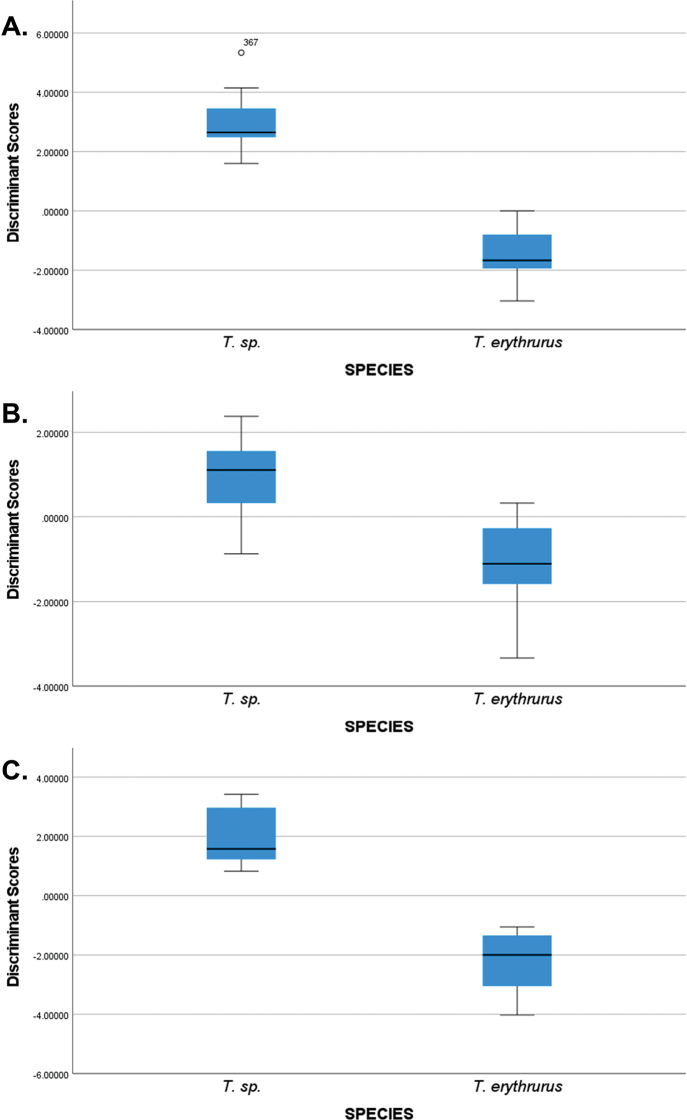
Box plots of discriminant scores classifying *T.erythrurus* and *T.* sp. **A** males with three variables entered (VEN, SC, and BSCK) achieved 100% success in discrimination and cross-validation **B** females with two variables entered (BSCK and SL) with lower discrimination success **C** females with an increased number of included variables (BSCK, SL, KTEMP, VS%25–23, HW1) increased discrimination success but decreased cross-validation success.

Discriminant analysis of males *T.erythrurus* and *T.* sp. resulted in 100% correct classification and cross-validation (in which each case is classified according to the functions generated by all cases other than that case) with three variables entered: VEN, SC, and BSCK (Fig. 2A). In females, BSCK and SL were entered but discrimination was less successful, with only 90.9% and 83.3% of *T.* sp. and *T.erythrurus* respectively being accurately classified (Fig. 2B). Increasing the number of included variables by entering them together rather than in a stepwise manner increased the accuracy of classification to 100% when using all cases, but cross-validation accuracy was reduced to 54.5% and 60% respectively (Fig. 2C). In this analysis, in addition to BSCK and SL, KTEMP, VS%25–23, and HW1 also contributed to the discrimination to a similar degree.

### ﻿Systematics

Evidence supporting the distinction of *Trimeresurus* sp. as a unique and independent lineage warranting species recognition was demonstrated in Chan et al. (2023) based on robust analyses of genomic data and is further substantiated by the morphological analysis presented above. Below, we provide a morphological description of the new species.

#### ﻿ Trimeresurus ayeyarwadyensis sp. nov.

Taxon classificationAnimaliaSquamataViperidae

CD221E78-F0D9-55C2-BF0C-BA4171F17208

https://zoobank.org/26B634FE-3296-4227-9F46-A9942137BE75

**Figure 3.**
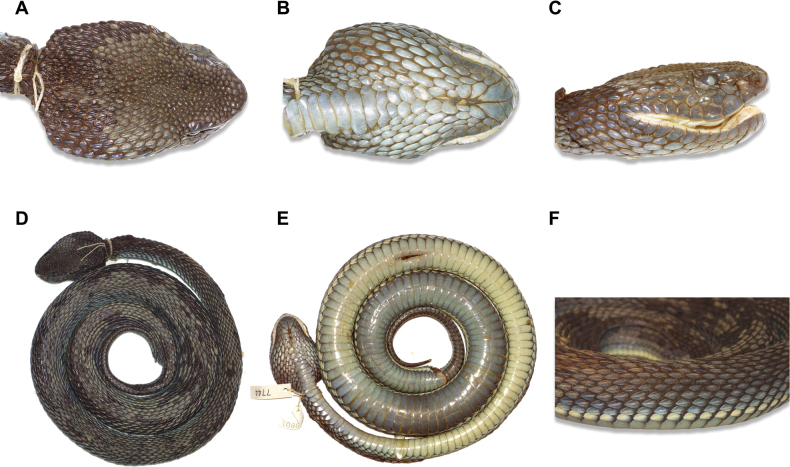
Holotype of *Trimeresurusayeyarwadyensis* sp. nov. (CAS 219801), adult female from Pyapon District, Ayeyarwady Division, Myanmar **A** dorsal view of head **B** ventral view of head **C** lateral view of right side of head **D** dorsal view of whole body **E** ventral view of whole body **F** lateral view of mid-body. Photographs by A. Malhotra.

Fig. 3A–F Vernacular name: Ayeyarwady pit viper

##### Type material.

***Holotype*.**CAS 219801, adult female, collected on 19 Jan. 2001 from Ayeyarwady Region, Pyapon District, Bogalay Township, Mein Ma Hla Kyun Wildlife Sanctuary, Mi Gyaung Gaung Pok Camp (16°00'45.1"N, 95°19'30.6"E) at 2025 hrs by H. Win, T. Thin, K.S. Lwin, A.K. Shein, and H. Tun.

***Paratypes*.**CAS 213587, adult male, collected on 9 Jan. 2000 from Yangon Region, Hlaw Ga Park, Mingalardon Township (17°1'36.5"N, 96°5'49.4"E) at 0800 hrs by H. Win, T. Thin, S.L. Oo, and S.W. Kyi; CAS 212245, adult female, collected on 22 Apr. 2000 from Ayeyarwady Region, vicinity of Mwe Hauk Village (16°16'34.3"N, 94°45'1.3"E) at 1935 hrs by J.B. Slowinski, G.R. Zug, R.S. Lucas, and J.V. Vindum; CAS 219783, adult male, collected on 18 Jan 2001 from Ayeyarwady Region, Pyapon District, Bogalay Township, Mein Ma Hla Kyun Wildlife Sanctuary, West Htaw Pai Camp (15°56'43.7"N, 95°19'2"E) at 2105 hrs by H. Win, T. Thin, K.S. Lwin, A.K. Shein, and H. Tun.

##### Diagnosis.

*Trimeresurus* can be distinguished from all other Asian pit-vipers by the condition of the first infralabial and nasal scale, which are at least partially fused. The new species can be diagnosed from other species of *Trimeresurus* by the following combination of characters: in both sexes (*n* = 19), 23–25 dorsal mid-body scale rows (mean 24.3 ± 0.97), 17 scale rows just anterior to vent (rarely 15 or 16) and body scales distinctly and sharply keeled; in males (*n* = 8), 150–170 ventral scales (mean 160.6 ± 5.6), 71–76 subcaudal scales (mean 73 ± 1.8); a minimum of 9–11 scales between supraoculars (mean 9.9 ± 0.6); between 5–9 scales touching the subocular scale (not counting pre- and post-oculars; mean 6.9 ± 0.9); supralabials 9–12 (mean 10.4 ± 0.8). In females (*n* = 11), 157–174 ventral scales (mean 165.1 ± 4.9), 52–58 subcaudal scales (mean 55 ± 2.1); a minimum of 10–12 scales between supraoculars (mean 10.8 ± 0.9); between 5–8 scales touching the subocular scale (not counting pre- and post-oculars; mean 6.5 ± 0.8); supralabials 10–12 (mean 10.9 ± 0.6).

##### Description of holotype

**(Fig. 3A–F).** This is a large female with a total length of 1008.3 mm (SVL 935 mm, TaL 148 mm), with indistinct darker dorsal markings and a distinct pale dorsolateral stripe covering 90% of the first scale row and extending onto the second scale row. The upper part of head lacking any paler color, and a postocular stripe is lacking. The ventral surface is paler but darkens towards the infralabials, which are the same color as the upper part of the head. Heavily keeled scales are present on body and head. Only a blunt keel is present on the first scale row adjacent to the ventrals at mid-body, but higher scale rows become progressively more sharply keeled with an obvious ridge at the center of the scale. Temporal scales and scales on the rear of the head are similarly sharply keeled, but scales between the supraoculars are tubercular rather than obviously keeled. Ventrals 161, subcaudals 54; there are 25 scale rows at 14 ventral scales, which is maintained until the 94^th^ ventral scale (thus there are 25 scale rows at mid-body) and reducing to 17 just anterior to the vent. There are 11 supralabials and 14 infralabials on the right side and 10 and 14, respectively, on the left side. There are a minimum of 10 tubercular scales between the supraoculars, with 16 between the inner rear edges of the supraoculars, which are relatively small and undivided. Internasals are separated by one scale; the first supralabial is almost completely fused with the scale surrounding the nostril, with only a small notch on its rear edge; there are 2 small scales between this fused scale and the fused second supralabial and loreal scale, which forms the anterior border of the pit; 1 or 2 scales are present between the supralabials and the subocular, which is bordered by 6 scales on the right and 7 scales on the left side of the head (not including the pre- and post-oculars). There are 2 postoculars and 8 paired chin shields, with the most anterior pair being the largest, between the first infralabials (which meet on the ventral side of the head) and the first ventral (defined as the first undivided scale on the ventral side of the head). The anal scale is entire, and the subcaudals are paired. The upper margin of the rostral scale is 39% the length of the lower margin.

##### Variation in the paratypes.

In CAS 213587, a male, the lateral white stripe is very prominent, and there is a faint postocular streak. It has a more uniformly colored ventral surface, which is closer to the shade of the dorsal surface, and continues on the head as far as the postocular streak, whereas in the second male, CAS 219783, the head is almost uniformly dark in coloration, being only slightly paler steel gray on the ventral surface and the supralabials. In CAS 212245 (a female), the ventral surface of body and head is distinctly paler, with this paler ventral color continuing onto the infra- and supralabials, but more patchy on the latter, and also appearing in patches on two to three scale rows above the supralabials. The internasals are in contact in both CAS 212245 and CAS 213587 but separated by one scale in CAS 219783. Ventrals are 170 in both CAS 213587 and CAS 212245 but 160 in CAS 219783; subcaudals 72 and 76 in the males and 57 in the female. Supralabials are 10/10 and 11/11 in CAS 213587 and CAS 219783, respectively, and 12/12 in the female CAS 212245, while the corresponding sublabial counts are 11/12, 12/13, and 14/14. While CAS 213587 and CAS 219783 have an almost entirely fused first supralabial and nasal, like the holotype, they lack any scales between this and the fused second supralabial/loreal scale. This scale is also fused only on the right side in CAS 213587. On the other hand, CAS 212245 has two scales between the fused first supralabial and nasal and the fused second supralabial/loreal scale as in the holotype, but the fusion of the first supralabial and nasal scale is only partial, with deep sutures extending towards the center from both sides, leaving only the area immediately below the nostril fused. The head is relatively smaller and narrower in the males, with only 10 (smallest number) and 13 or 14 (between rear edges) cephalic scales between the supraoculars (compared to 12 and 16 in the female, and fewer paired chin shields (6 or 7 compared to 8 in the females). There are 23 scale rows at mid-body and 15 scale rows anterior to the vent in CAS 213587, but 25/17 in the other two specimens.

The hemipenis is everted on both sides of the male paratype CAS 219783 and is similar to other species of *Trimeresurus*, being elongated and bifurcated for about 75–80% of the total length and lacking basal spines. The post-fork region is calcyed, and the edges of the calyces are pointed and longer near the fork. The insertion of the hemipenis retractor muscle is at 75% of the length of the tail measured from the vent.

##### Comparisons.

*Trimeresurusayeyarwadyensis* sp. nov. is most closely related to *T.purpureomaculatus* and *T.erythrurus* and is also morphologically most similar to those species. They can be distinguished from other mainland *Trimeresurus* species (sensu Malhotra and Thorpe 2004a) in having 17 dorsal scale rows just anterior to the vent, compared to 15, and generally more than 21 scale rows at mid-body. *Trimeresuruspurpureomaculatus* can be distinguished from *T.ayeyarwadyensis* sp. nov. by a combination of a more triangular rostral scale and a larger number of cephalic scales between the supraoculars (averaging 15.5 at the widest point versus 13.15). *Trimeresuruserythrurus* is less easily distinguished but tends to be smaller (especially for females) and has less heavily and sharply keeled scales on the body and temporal region.

Dorsal color pattern is highly variable ranging from light or olive-green with no distinct blotches, similar to *T.erythrurus*, to olive-green with dark, irregular blotches, similar to *T.purpureomaculatus*. The ventrolateral side of head is yellowish, and there is a white dorsoventral stripe present in both sexes. The iris color varies from deep red to golden.

##### Distribution.

*Trimeresurusayeyarwadyensis* sp. nov. occurs at Hlawga Park in the Yangon Region and Pyapon and Myaungmya districts in the Ayeyarwady Region. The northern and western limits of its distribution likely lie somewhere in between the Myaugmya and Pathein districts in the Ayeyarwady Region. Southward, it could occur in mangroves in Mon State.

##### Natural history.

In the Pyapon and Myaungmya districts in the Ayeyarwady Region, snakes were found in mangrove forests, whereas at Hlawga Park in the Yangon Region, snakes were found in forested habitats around a lake that is not connected to any mangrove system. In that regard, *T.ayeyarwadyensis* sp. nov. is more similar to *T.erythrurus* as opposed to *T.purpureomaculatus*, which is a strict mangrove-associated species.

##### Etymology.

The specific epithet “*ayeyarwadyensis*” refers to the Ayeyarwady River (= Irrawaddy River), which is the largest and one of the most important rivers in Myanmar. The river forms an expansive delta that is bounded by the Pathein River to the west and the Yangon River to the east. These rivers and their associated basins also mark the westernmost and easternmost distribution boundaries of *T.ayeyarwadyensis* sp. nov.

## ﻿Discussion

**Figure 4.**
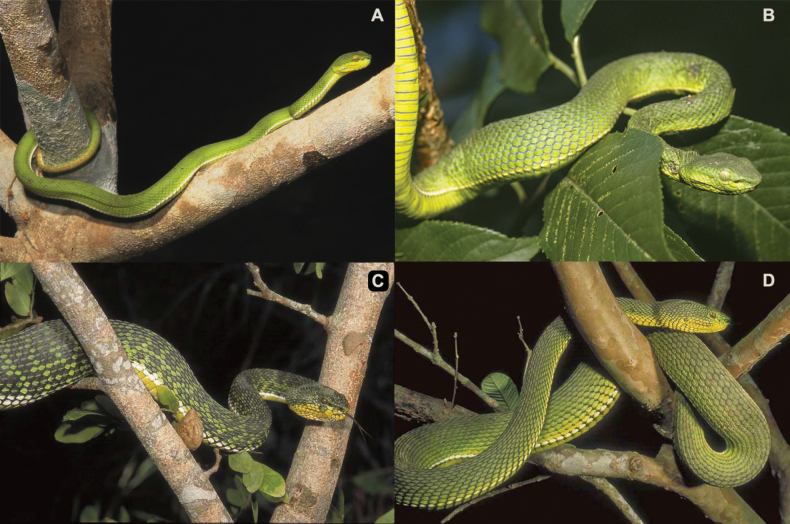
*Trimeresurus* species in Myanmar **A***T.ayeyarwadyensis* sp. nov. (CAS 213410) from Hlawga Wildlife Park, Yangon Region (photo by CAS-Myanmar Herpetology Survey team, CAS-MHS) **B***T.erythrurus* (CAS 235958) from Phalum District, Chin State (photo by Hla tun) **C***T.ayeyarwadyensis* sp. nov. (CAS 219764) from Meinmahla Kyun Wildlife Sanctuary, Pyapon District, Ayeyarwady Region (photo by Hla tun) **D***T.ayeyarwadyensis* sp. nov. (CAS 212245) from Mwe Hauk Village, Ayeyarwady Region (photo by Dong Lin).

**Figure 5.**
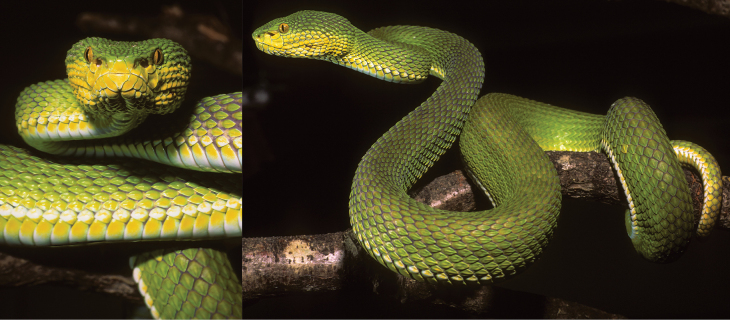
Photographs of an unvouchered, live specimen of *Trimeresurusayeyarwadyensis* sp. nov. from the Yangon Region, Myanmar (Photos by Wolfgang Wüster).

In terms of color pattern, *Trimeresurusayeyarwadyensis* sp. nov. shares characteristics with both *T.purpureomaculatus* and *T.erythrurus*. For example, specimen CAS 213410 from Yangon is bright green with no dorsal markings (Fig. 4A) and is virtually identical to *T.erythrurus* (Fig. 4B). A photograph of an unvouchered live specimen from Yangon corroborates this observation (Fig. 5). Contrastingly, CAS 219764 from Pyapon District in the Ayeyarwady Region has a dark or olive-green base dorsal coloration with distinct dark blotches (Fig. 4C) reminiscent of *T.purpureomaculatus*—albeit *T.purpureomaculatus* does not have a green base dorsal coloration (Chan et al. 2022). On the other hand, CAS 212245 from Myaungmya District in the Ayeyarwady Region appears to be an intermediate of the other two specimens in having a dark or olive-green base dorsal coloration with no distinct blotches (Fig. 4D). Despite these variations, all three specimens were unambiguously shown to represent a distinct, monophyletic lineage that is not conspecific with either *T.purpureomaculatus* or *T.erythrurus* (Chan et al. 2023). The distinct evolutionary trajectory of the new species coupled with the lack of morphological differentiation makes *T.ayeyarwadyensis* sp. nov. an archetypal example of a true cryptic species.

Chan et al. (2023) showed that both *T.erythrurus* and *T.ayeyarwadyensis* sp. nov. co-occur in the Ayeyarwady Region. A population in the Pathein District was shown to be highly admixed between *T.erythrurus* and *T.ayeyarwadyensis* sp. nov. but was clearly conspecific with *T.erythrurus*, which is reflected in the morphological similarity presented in this paper. A mere ~50 km away in the Myaungmya District, a relatively pure population of *T.ayeyarwadyensis* sp. nov. occurs instead of *T.erythrurus* (Chan et al. 2023), indicating that there is a narrow contact zone around this area that serves as a natural distribution barrier for both *T.erythrurus* and *T.ayeyarwadyensis* sp. nov. (*T.erythrurus* has not been documented south of Pathein and *T.ayeyarwadyensis* sp. nov. has not been documented north or west of Myaungmya). No live photographs are available of snakes from the Pathein District, but examination of preserved specimens shows a lack of distinct dorsal markings. Towards the south, there is a distribution gap at Mon State where neither *T.ayeyarwadyensis* sp. nov. nor *T.purpureomaculatus* has been documented. However, this could be due to the lack of sampling in that area. *Trimeresuruspurpureomaculatus* has been documented as far north as the Dawei District in the Tanintharyi Region and it is plausible that another contact zone exists between *T.ayeyarwadyensis* sp. nov. and *T.purpureomaculatus* in the intervening area, possibly within Mon State. Interestingly, the mtDNA placed populations from the Tanintharyi Region in a clade with the new species from Yangon and Areyearwady (Chan et al. 2022), whereas the genomic DNA placed the Tanintharyi populations in a clade with *T.purpureomaculatus* (Chan et al. 2023), thus reflecting putative hybridization or possible mtDNA introgression in this group. The demographic history and phylogeographic patterns of *T.erythrurus*, *T.ayeyarwadyensis* sp. nov., and *T.purpureomaculatus* represent a compelling model to study speciation dynamics at contact zones and allude to the possibility of similar phenomena occurring in other groups of Asian pit-vipers. Our results provide a useful roadmap to guide future studies and highlight the need for targeted sampling in the Ayeyarwady Region and Mon State.

## Supplementary Material

XML Treatment for Trimeresurus ayeyarwadyensis

## ﻿Additional information

### Author ORCIDs

Kin Onn Chan https://orcid.org/0000-0001-6270-0983

Shahrul Anuar https://orcid.org/0000-0003-0648-7318

Ananthanarayanan Sankar https://orcid.org/0000-0002-2116-5299

Ingg Thong Law https://orcid.org/0009-0002-6699-6587

Ing Sind Law https://orcid.org/0000-0002-0727-2944

Rasu Shivaram https://orcid.org/0009-0005-6839-3474

Ching Christian https://orcid.org/0000-0001-6972-6836

Daniel G. Mulcahy https://orcid.org/0000-0001-6614-2405

Anita Malhotra https://orcid.org/0000-0002-1738-9046

### Data availability

All of the data that support the findings of this study are available in the main text or Supplementary Information.

## Supplementary materials

Supplementary material 1 Raw morphological data of specimens examined in this study Data type xlsx https://binary.pensoft.net/file/952202This dataset is made available under the Open Database License (http://opendatacommons.org/licenses/odbl/1.0/). The Open Database License (ODbL) is a license agreement intended to allow users to freely share, modify, and use this Dataset while maintaining this same freedom for others, provided that the original source and author(s) are credited.Kin Onn Chan, Shahrul Anuar, Ananthanarayanan Sankar, Ingg Thong Law, Ing Sind Law, Rasu Shivaram, Ching Christian, Daniel G. Mulcahy, Anita Malhotra
